# Structural Characteristics and Anticoagulant Property In Vitro and In Vivo of a Seaweed Sulfated Rhamnan

**DOI:** 10.3390/md16070243

**Published:** 2018-07-20

**Authors:** Xue Liu, Shuyao Wang, Sujian Cao, Xiaoxi He, Ling Qin, Meijia He, Yajing Yang, Jiejie Hao, Wenjun Mao

**Affiliations:** 1 Key Laboratory of Marine Drugs of Ministry of Education, Shandong Provincial Key Laboratory of Glycoscience and Glycotechnology, School of Medicine and Pharmacy, Ocean University of China, Qingdao 266003, China; wenshan389161833@163.com (X.L.); wangshuyao@stu.ouc.edu.cn (S.W.); caosujian@stu.ouc.edu.cn (S.C.); hexiaoxi@ouc.edu.cn (X.H.); ql1599@stu.ouc.edu.cn (L.Q.); hmj@stu.ouc.edu.cn (M.H.); yangyajing@stu.ouc.edu.cn (Y.Y.); 2009haojie@ouc.edu.cn (J.H.); 2 Laboratory for Marine Drugs and Bioproducts of Qingdao National Laboratory for Marine Science and Technology, Qingdao 266237, China; 3 Biology Institute, Qilu University of Technology (Shandong Academy of Sciences), Jinan 250103, China

**Keywords:** seaweed polysaccharide, anticoagulant property, fibrin(ogen)olytic activity, thrombolytic activity

## Abstract

Great diversity and metabolite complexity of seaweeds offer a unique and exclusive source of renewable drug molecules. Polysaccharide from seaweed has potential as a promising candidate for marine drug development. In the present study, seaweed polysaccharide (SPm) was isolated from *Monostroma angicava*, the polymeric repeat units and anticoagulant property in vitro and in vivo of SPm were investigated. SPm was a sulfated polysaccharide which was mainly constituted by 3-linked, 2-linked-α-l-rhamnose residues with partially sulfate groups at C-2 of 3-linked α-l-rhamnose residues and C-3 of 2-linked α-l-rhamnose residues. Small amounts of xylose and glucuronic acid exist in the forms of β-d-Xyl*p*(4SO_4_)-(1→ and β-d-GlcA-(1→. SPm effectively prolonged clotting time as evaluated by the activated partial thromboplastin time and thrombin time assays, and exhibited strong anticoagulant activity in vitro and in vivo. The fibrin(ogen)olytic and thrombolytic properties of SPm were evaluated by plasminogen activator inhibitior-1, fibrin degradation products, D-dimer and clot lytic rate assays using rats plasma, and the results showed that SPm possessed high fibrin(ogen)olytic and thrombolytic properties. These results suggested that SPm has potential as a novel anticoagulant agent.

## 1. Introduction

Thrombotic diseases are reported to contribute to 30% early deaths globally [1,2]. Anticoagulant drugs have been extensively used as an adjunct therapy in thrombotic diseases, and heparin is now the initial choice. Heparin has adverse events, including development of thrombocytopenia, hemorrhagic effect, and ineffectiveness in congenital or acquired anthrombin deficiencies [3,4]. In addition, heparin is mostly extracted from pig intestine or bovine lung, where it occurs in low concentrations. Furthermore, the incidenc of prion-related diseases in mammals and the increasing requirement of anticoagulant therapy demonstrate that it is necessary to look for alterative sources of anticoagulant agents [5,6,7,8].

Seaweeds are a huge source of natural products owing to their specific marine environment [9,10]. Among these, polysaccharides occupy a preeminent position and have high potential as preventing and therapeutic agents against several diseases, for their anticancer, anti-inflammatory, antiviral, antibacterial and anticoagulant properties [11,12,13,14]. Many polysaccharides from seaweeds are already used in the food industry, including alginates, carrageenans and fucoidans [11,15,16,17,18,19]. Polysaccharides from seaweeds are only partly explored, due to great diversity and metabolite complexity of seaweeds offer a unique and exclusive source of active polysaccharides.

It is noteworthy that seaweed could produce specific sulfated polysaccharides which are mainly composed of α-l-rhamnose moiety. The distribution of sulfated rhamnan composed of large amounts of α-l-rhamnose is quite limited in the nature, though single α-l-rhamnose is widely distributed in plants and microorganism [20,21,22]. So far a few limited reports on structure and biological activity of the sulfated rhamnan from seaweed have been demonstrated [22,23,24,25,26,27].

*Monostroma angicava* is widely distributed through the World’s seas, analysis of this species is very important in pharmaceutical applications. In the present study, a novel sulfated rhamnan was obtained from seaweed *M. angicava*, its structural characteristics and anticoagulant property in vitro and in vivo were investigated. The seaweed polysaccharide has potential as a drug or a food supplement for health promotion and treatment of cardiovascular disease. The detailed results of our analyses are presented in the paper.

## 2. Results and Discussion

### 2.1. Structure Elucidation of the Seaweed Polysaccharide

Seaweed polysaccharide (SPm) was isolated from *M. angicava* by water extraction and purified by Q Sepharose Fast Flow and Sephacryl S-400/HR column. High performance gel permeation chromatography (HPGPC) analysis of SPm gave a homogeneous profile with molecular weight of 91.9 kDa (Figure S1a). SPm presented high sulfate content (30.18%) besides uronic acid (8.26%). High performance liquid chromatography (HPLC) analysis showed that SPm contained rhamnose as the major sugar (85.60 mol%), together with xylose (6.39 mol%) and glucuronic acid (8.01 mol%) (Figure S1b). Reverse-phase HPLC analysis showed that rhamnose had the l-configuration, while glucuronic acid and xylose had the d-configuration.

Fourier-transform infrared (FTIR) spectrum of SPm showed absorption of high intensity at 1240 cm^−1^ related to sulfate groups (stretching vibration of S–O of sulfate) together with another band at 855 cm^−1^. The absorption at 855 cm^−1^ derived from stretching vibration of C–O–S of sulfate in axial position (Figure S1c). The intense and broad band at 3445 cm^–1^ was due to the stretch vibration of hydroxyl groups, and the signal at 2940 cm^–1^ was assigned to the stretch vibration of the C–H bond. In addition, the band at 1630 cm^–^^1^ was due to asymmetric stretching vibration of COO^−^ of uronic acids. The band at 1452 cm^–^^1^ was the absorption peak of variable angle vibration of C–H bond. The band at 1051 cm^–^^1^ was from the stretching vibration of C–O. 

Methylation analysis showed that SPm mainly consisted of (1→2)-linked, (1→3)-linked, (1→2,3)-linked rhamnose with minor amount of (1→4)-linked-xylose units (Table S1). After desulfation of SPm, the increase of (1→2)-linked and (1→3)-linked rhamnose residues, along with the decrease of (1→2,3)-linked rhamnose residues, showed that SPm contained (1→3)-linked rhamnose residues with sulfation at C-2 and (1→2)-linked-rhamnose residues with sulfation at C-3. Furthermore, (1→)-linked xylose appeared as a substitute of (1→4)-linked xylose in SPm-Ds which indicated that the sulfation site was at the C-4 of (1→)-linked xylose. To confirm the linkage pattern of glucoronic acid, methylation analysis was also carried out with the carboxyl-reduced polysaccharide (SPm-R) and the carboxyl-reduced and desulfated polysaccharide (SPm-RDs). Equal amount of (1→)-linked glucose was found in SPm-R and SPm-RDs, indicating the glucuronic acid in SPm existed in the form of (1→)-linked glucuronic acid residue. It could be deduced that 27.38% of total number of the rhamnose in SPm was substituted by sulfate ester groups, specifically to (1→3)-linked rhamnose was about 15.90%, and to (1→2)-linked rhamnose was about 11.48%. The xylose was sulfated at the C-4.

In the ^1^H NMR spectrum of SPm (Figure S2a), five anomeric proton signals at 5.49, 5.34, 5.29, 5.26 and 5.08 ppm assigned to the α-rhamnopyranose residues were observed which had relative integrals of 1.0: 0.71: 0.38: 1.49: 2.28, and were labeled A, B, C, D and E, respectively. The signal appearing at 1.38 ppm was the proton of CH_3_ group of rhamnose residues. In the anomeric region of ^13^C NMR spectrum (Figure S2b), two major signals occurred at 100.74 ppm and 103.32 ppm. The signal at 105.93 ppm could arise from anomeric carbon signals from β-d-xylosyl units as well as β-d-glucuronic acid residues [28]. The signal at 18.26 ppm was attributed to the CH_3_ of the rhamnose residues.

According to the sugar composition, methylation analyses and the data offered by ^1^H NMR, ^13^C NMR, ^1^H–^1^H COSY (Figure S2c), ^1^H–^13^C HSQC (Figure S2d) and ^1^H–^1^H NOESY (Figure S2e) spectra, the sugar residues could be assigned as A →3)-α-l-Rha*p*(2SO_4_)-(1→, B →2)-α-l-Rha*p*(3SO_4_)-(1→, C →2,3)-α-l-Rha*p*-(1→, D →2)-α-l-Rha*p*-(1→ and E →3)-α-l-Rha*p*-(1→, respectively. In particularly, the signals H-3/H-2(4.72/4.55 ppm) with down-field shifts of residues A and B were due to the sulfation at C-3 and C-2, respectively. ^1^H and ^13^C chemical shifts of SPm are listed in Table S2.

**Figure 1:**
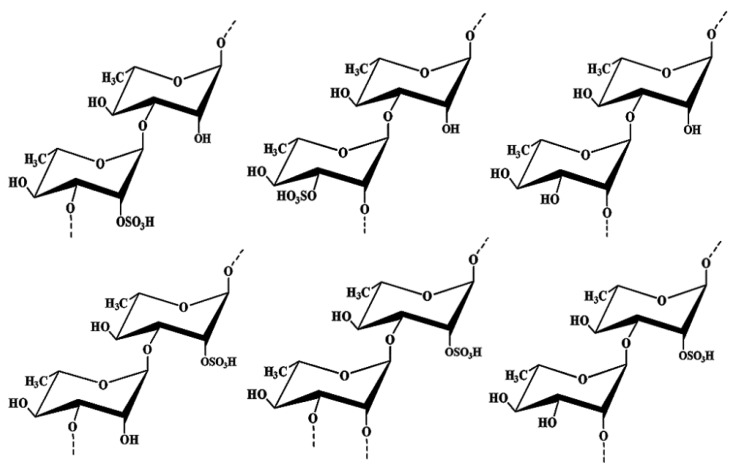
Structures of the main repeating disaccharides of SPm.

From the ^1^H–^1^H NOESY spectrum of SPm, the anomeric proton signals of A, B and D were correlated with the H-3 of E and indicated the sequences →3)-α-l-Rha*p*(2SO_4_)-(1→3)-α-l-Rha*p*-(1→, →2)-α-l-Rha*p*(3SO_4_)-(1→3)-α-l-Rha*p*-(1→ and →2)-α-l-Rha*p*-(1→3)-α-l-Rha*p*-(1→. Moreover, the anomeric proton signals of C and D were correlated with the H-3 of A which indicated the sequences →2,3)-α-l-Rha*p*-(1→3)-α-l-Rha*p*(2SO_4_)-(1→ and →2)-α-l-Rha*p*-(1→3)-α-l-Rha*p*(2SO_4_)-(1→. Sequence →3)-α-l-Rha*p*-(1→3)-α-l-Rha*p*(2SO_4_)-(1→ was deduced by the cross signal H-1(E)/H-3(A). Structures of the main repeating disaccharides were shown in Figure 1.

The seaweed polysaccharide SPm had different structure from other sulfated rhamnans from seaweed [25,26,29,30,31]. The sulfate substitutions were located at the C-2 of →3)-α-l-Rha*p*-(1→, C-3 of →2)-α-l-Rha*p*-(1→ and C-4 of β-d-Xyl*p*-(1→, besides glucuronic acid existed only in the form of β-d-GlcA-(1→. Especially, the terminal β-d-xylosyl 4-sulfate and β-d-glucuronic acid residues were infrequently found in the sulfated rhamnans from Monostromaceae species [25,26,29,30,31]. Algae from the genera *Ulva* and *Enteromorpha* produced rhamnan-type sulfated polysaccharides which are mainly composed of 4-linked rhmanose 3-sulfate and 4-linked glucuronic or iduronic acid residues [10]. Hence, the glycosidic linkage and sulfation patterns of SPm are distinctly different from the sulfated rhamnans observed for other green algae belonging to genera *Ulva* and *Enteromorpha*.

### 2.2. Anticoagulant Activity In Vitro and In Vivo of SPm

**Figure 2:**
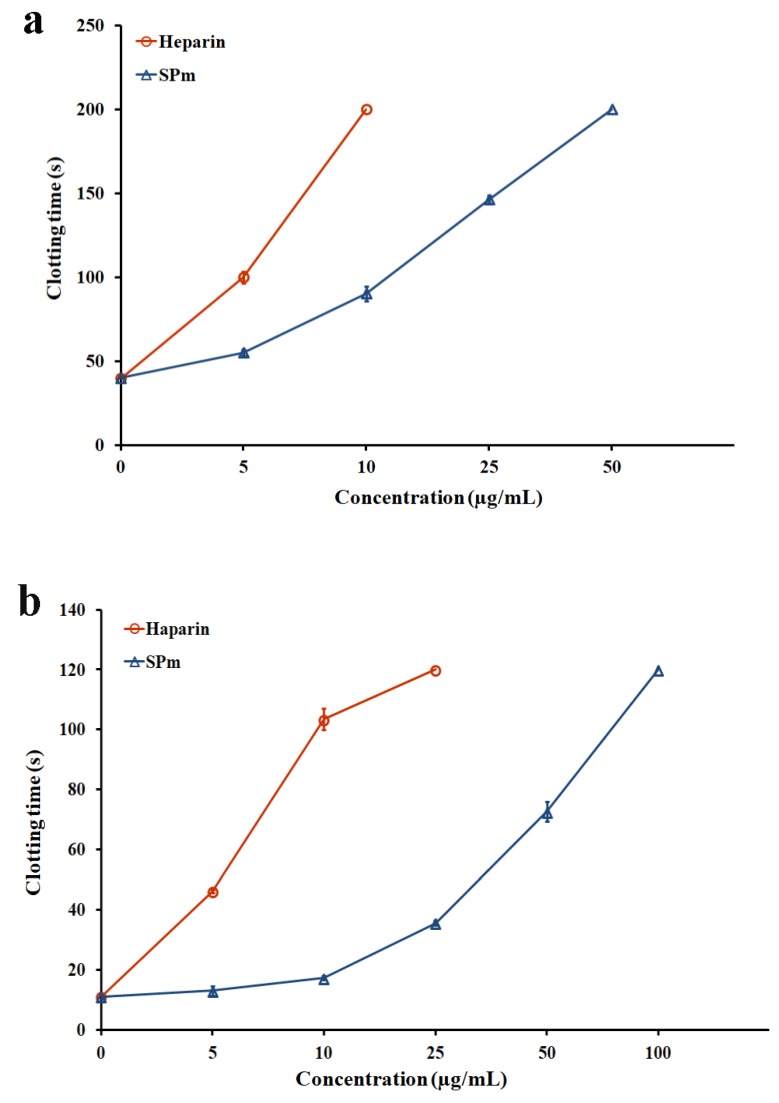

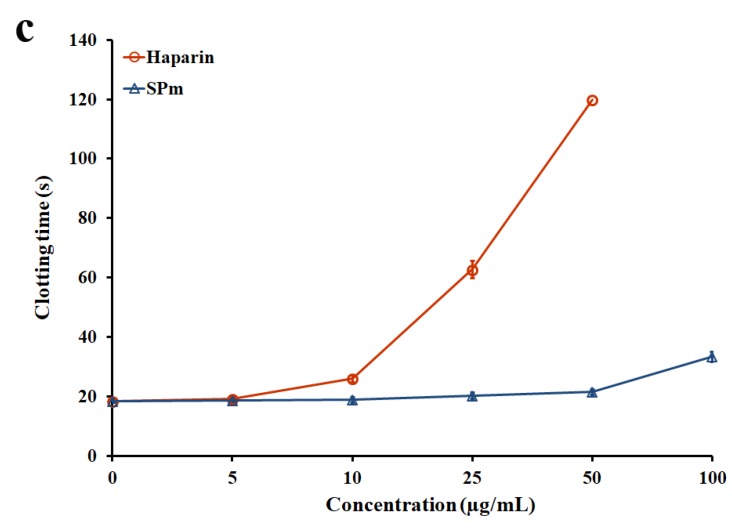
Anticoagulant activity in vitro of SPm. (**a**) APTT; (**b**) TT; (**c**) PT. 33.88% of total number of the sugar residues in SPm was substituted by sulfate ester groups.

Anticoagulant activity in vitro was evaluated by assays of activated partial thromboplastin time (APTT), prothrombin time (PT) and thrombin time (TT) using heparin as a reference. As listed in Figure 2a–c, the APTT activity of SPm slowly increased with increasing concentration of the polysaccharide, and the signal for clotting time was up to 200 s at 50 µg/mL. APTT reflects the integrity of the endogenous and/or common pathways of the procoagulant cascade (VIII, IX, XI). SPm also effectively prolonged the TT, and the signal for clotting time was more than 120 s at 100 µg/mL. The prolongation of TT demonstrated the inhibition of thrombin activity or fibrin polymerization. However, the effect of SPm on PT was markedly different from that of heparin, and lack of prolongation effect of SPm on PT was discovered. PT is a sensitive screening test for the extrinsic coagulation pathway. The results demonstrated that SPm had a high anticoagulant property in vitro which inhibited both the intrinsic and/or common pathways of coagulation and thrombin activity or conversion of fibrinogen to fibrin. The anticoagulant activity of SPm was different from that of heparin. It was observed that the APTT activity by heparin quickly increased and clotting time was more than 200 s at 10 µg/mL. The TT activity by heparin also rapidly increased and the clotting time was more than 120 s at 25 µg/mL.

**Figure 3:**
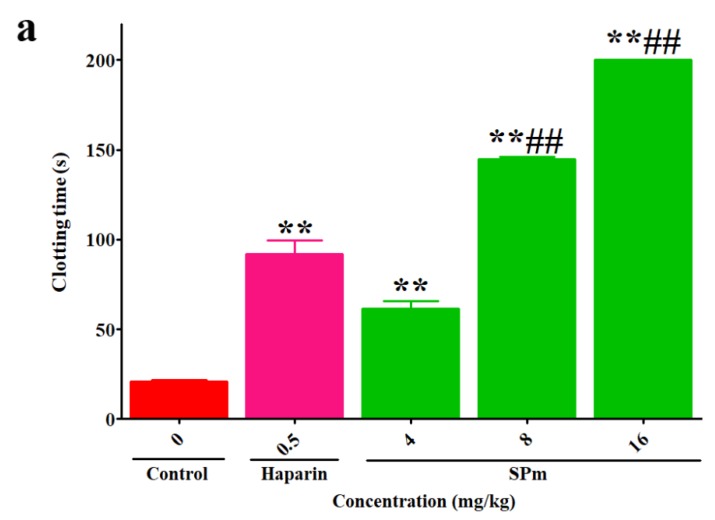

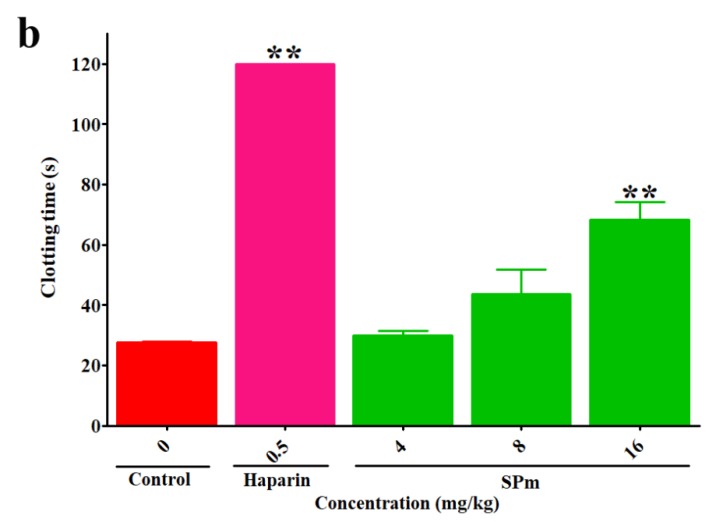
Anticoagulant activity in vivo of SPm. (**a**) APTT; (**b**) TT. Significance: ** *p* < 0.01 vs. the control group; ^##^ *p* < 0.01 vs. the heparin group. 33.88% of total number of the sugar residues in SPm was substituted by sulfate ester groups.

Anticoagulant activity of SPm in vivo was also evaluated by APTT, TT and PT assays using heparin as a reference. As shown in Figure 3a,b, the anticoagulant activity of SPm was also concentration-dependent. APTT was strongly prolonged by SPm with increasing concentration of the polysaccharide and clotting time was more than 200 s at 16 mg/kg. Moreover, the TT activity by SPm increased with increasing concentration of the polysaccharide. The lack of prolongation effect of SPm on PT was discovered (data not shown). It was noted that the prolongation effects of SPm at 8 and 16 mg/kg on the APTT activity were significantly higher than that of heparin in the concentration used in the experiment. However, the prolongation effect of SPm on the TT activity was weaker than that of heparin.

SPm exhibited a higher anticoagulant activity in vitro than the sulfated rhamnans P, WF1 and Ls2-2 from *Monostroma* species [26,32,33]. P from *M. latissimum* typically included 1,3-, 1,2-, 1,2,3-, 1,2,3,4- and 1,2,4-linked rhamnose [32]. WF1 from *M. nitidum* mainly contained →2)-α-l-Rha*p*-(1→ and →4)-α-l-Rha*p*-(1→ residues with sulfate substitutions at C-3 /C-4 of →2)-α-l-Rha*p*-(1→ residues [33]. Ls2-2 from *M. angicava* Kjellm possessed the branches consisting of rhamnose and glucuronic acid residues, and the glucuronic acid was at C-3 and/or C-2 of non-reducing terminal rhamnose residues [26]. In addition, it is interesting to note that the anticoagulant activity of SPm was also higher than that of the sulfated rhamnan PF2 from *M. angicava* which consisted of only rhamnose with sulfate ester at C-3 of →2)-α-l-Rha*p*-(1→ residues [25]. However, SPm had a lower anticoagulant activity than the sulfated rhamnan from *M. nitidum* [34] and PML from *M. latissimum* [30], though they mainly contained →2)-α-l-Rha*p*-(1→ and →3)-α-l-Rha*p*-(1→ residues. The sulfate ester of the polysaccharide from *M. nitidum* was substituted at C-2 of →3)-α-l-Rha*p*-(1→ residue [34], while the sulfate groups of PML were located at C-2 of →3)-α-l-Rha*p*-(1→ and C-3 of →2)-α-l-Rha*p*-(1→ residues [30]. The results suggested that rhamnan-type sulfated polysaccharides express anticoagulant activity not merely as a function of charge density. The structural basis of this activity positively depends on the monosaccharide composition, sites of sulfation and/or of the glycosidic linkage, and also on the proportion and/or the distribution of sulfation pattern [17].

### 2.3. Fibrin(ogen)olytic Activity of SPm

Fibrin(ogen)olytic activity was evaluated by assays of D-dimer, plasminogen activator inhibitior-1 (PAI-1) and fibrin(ogen) degradation products (FDP) using urokinase as a reference (Table 1). D-dimer is a fibrin degradation product which is produced from crosslinked fibrin by the action of plasmin. D-dimer contains two crosslinked D fragments of the fibrin protein [35]. Compared with the control group, the level of D-dimer was obviously increased by SPm. The increasing effects of SPm at 8 mg/kg and 16 mg/kg on the level of D-dimer were significantly higher than that of urokinase in the concentration used in the experiment, indicating that SPm had good fibrinolytic and thrombolytic activity. PAI-1 is a primary regulator of the fibrinolytic system which inhibited the effects of tissue type plasminogen activator [36]. Compared with the control group, the level of PAI-1 was effectively decreased by SPm. Moreover, the decreasing effects of SPm at 8 mg/kg and 16 mg/kg on PAI-1 were significantly higher than that of urokinase. FDP is small protein fragment present in the blood after a blood clot is degraded by fibrinolysis. Compared with the control group, the treatment with SPm resulted in significant increasing the level of FDP. The increasing effects of SPm at 8 mg/kg and 16 mg/kg on FDP were markedly higher than that of urokinase. These results suggested that SPm had a high fibrin(ogen)olytic activity.

A few limited reports on the fibrin(ogen)olytic activity of the sulfated rhamnans have been presented [25,26]. It was observed that the decreasing effect of SPm on PAI-1 was stronger than that of PF2 and Ls2-2. Moreover, the increasing effects of SPm on FDP and D-dimer levels were higher than those of PF2 and Ls2-2. The structural characteristics of SPm were different from those of Ls2-2 and PF2 though they were mainly composed of →3)-α-l-Rha*p*-(1→ and/or →2)-α-l-Rha*p*-(1→ residues. Further work is required to investigate the relationship between the fine structure and fibrin(ogen)olytic activity of sulfated rhamnans with different structure.

### 2.4. Thrombolytic Activity of SPm

The thrombolytic activity in vitro of SPm was evaluated by assay of clot lytic rate using urokinase as a reference. As shown in Table 2, the clot lytic rate of SPm was markedly higher than that of control. Moreover, it was observed that the clot lytic rates of SPm at 8, 16 mg/mL were significantly higher than that of urokinase in the concentration used in the experiment. The data suggested that SPm had a high thrombolytic activity in vitro. It was noted that SPm had a higher clot lytic rate in vitro than PF2 and Ls2-2 [25,26]. SPm was a rhamnan-type sulfated polysaccharide differing from previously described sulfated rhamnans Ls2-2 and PF2. Comparison of the thrombolytic activity of the polysaccharides indicated that complex relationships existed between the structure and thrombolytic property of the polysaccharides. The differences of thrombolytic activity may be attributed to the structural feature variations of the polysaccharides.

With regards to the increasing incidence of thrombotic diseases, effective drugs or functional foods are urgently needed. Marine algae synthesize a great variety of water-soluble polysaccharides. Most of them are sulfated and have various biological activities. Some excellent reviews describing the structure, properties and applications of seaweed polysaccharides have been published [37,38,39]. Over the last few decades, the functional food and pharmaceutical industries have shown a strong interest in polysaccharides derived from marine algae. Algal anticoagulant polysaccharides hold great promise as a potential instead source of heparin from animal sources for therapy, as they are considered safer than heparin because of being free of the bovine spongiform encephalitis problem. The present results showed that SPm possessed strong anticoagulant activity in vitro and in vivo, and also exhibited high fibrin(ogen)olytic and thrombolytic activities. The seaweed polysaccharide may be a promising anticoagulant polysaccharide and has potential as a drug or a food supplement for health promotion and treatment of cardiovascular disease. Further studies in the anticoagulant seaweed polysaccharide are required.

## 3. Materials and Methods

### 3.1. Materials

Seaweed *M. angicava* was collected from the coast of Qingdao, China on April 2013, which is in growth mature period of the seaweed. The raw material was thoroughly washed with tap water, air-dried and stored at room temperature in a dry environment. l-rhamnose, l-arabinose, d-xylose, l-fucose, d-mannose, d-galactose, d-glucose, d-glucuronic acid, d-galacturonic acid, d-glucosamine and heparin were from Sigma-Aldrich Chemical Co. (St. Louis, MO, USA). Pullulan standards (*Mw*: 21.1, 47.1, 107, 200, 344, and 708 kDa) were from Showa Denko K.K. (Tokyo, Japan). APTT assay reagent (ellagic acid + bovine phospholipids reagent), TT assay reagent (bovine thrombin) and PT assay reagent (rabbit thromboplastin) were from Nanjing Jiancheng Bioengineering Institute (Nanjing, China). PAI-1 and D-dimer kits were from Simens Healthcare Diagnostics Products (Marburg, Germany). FDP kit was from BIOLINKS CO., LTD. (Tokyo, Japan).

### 3.2. Animals

Male Sprague-Dawley rats (220–250 g body weight) were housed at 23 ± 2 °C under 12 h light and dark cycles and were provided access to food and water *ad libitum*. Anticoagulant property in vivo was performed by assays of APTT, TT and PT. Fibrin(ogen)olytic in vivo property was evaluated by assays of D-dimer, PAI-1 and FDP. The male Sprague-Dawley rats were randomly divided into six experimental groups (10 rats/group). The experimental rats were anaesthetized with 15% urethane, and then injected with SPm, heparin or urokinase. Saline solution was used as control. The experiments were performed in accordance with the Guidelines of Animal Ethics Committee of Ocean University of China.

### 3.3. Extraction and Purification of the Sulfated Polysaccharide

The milled alga (80 g) was extracted with distilled water (1:30 alga: water) at room temperature for 3 h. After centrifugation, the resulting precipitation was homogenized with distilled water (1: 30 alga: water), and extracted at 100 °C for 3 h. After cooling to the room temperature, the supernatant was collected by centrifugation (3600× *g*, 10 min), concentrated, and dialyzed in a cellulose membrane (molecular weight cut-off 3500) against distilled water at room temperature for three successive days. The retained fraction was recovered, concentrated by rotary evaporation, precipitated with 95% ethanol (4 vols) and dried at 40 °C. The crude polysaccharide (15.06 g) was dissolved in water, loaded onto a Q Sepharose Fast Flow column (30 cm × 3.5 cm, GE Healthcare Life Sciences, Piscataway, NJ, USA), and eluted with a step-wise gradient of 0, 0.5, 1.5, 2.5, 3.0 mol/L NaCl at a flow rate of 1 mL/min. Eluate was collected by auto-fraction collector (6 mL/tube, Model 2110, Bio-Rad). Total sugar content of the eluate was determined by the phenol-sulfuric acid method. The fractions eluted by 1.5 mol/L NaCl was combined, dialyzed, loaded onto a Sephacryl S-400/HR column (100 cm × 2.5 cm; GE Healthcare Life Sciences, Piscataway, NJ, USA), and eluted with 0.2 mol/L NH_4_HCO_3_ at a flow rate of 0.3 mL/min. After these steps, a purified sulfated polysaccharide was obtained, and named as SPm (1.68 g).

### 3.4. Composition Analysis

The assay of purity and molecular weight of polysaccharide was performed on an Agilent 1260 Infinity HPLC instrument (Agilent Technologies Co. Ltd., Palo Alto, CA, USA) fitted with a Agilent RID-10A Series refractive index detector (Agilent Technologies Co. Ltd., Palo Alto, CA, USA) and using an a Shodex OHpak SB-804 HQ column (8.0 mm × 300 mm, Showa Denko K.K., Tokyo, Japan) [25]. The molecular weight was estimated by reference to a calibration curve made by pullulan standards (*Mw*: 21.1, 47.1, 107, 200, 344, and 708 kDa, Showa Denko K.K., Tokyo, Japan). Sulfate ester content was estimated according to the method of Therho and Hartiala [40]. Uronic acid content was determined by the carbazole-sulfuric acid method [41]. Total sugar content was determined by the phenol-sulfuric acid method using rhamnose as the standard [42]. Protein content was determined according to the method of Bradford using bovine serum albumin as the standard [43]. Monosaccharide composition was performed on an Agilent 1260 Infinity HPLC instrument (Agilent Technologies Co. Ltd., Palo Alto, CA, USA) fitted with an Agilent XDB-UV detector (Agilent Technologies Co. Ltd., Palo Alto, CA, USA) and using an Eclipse XDB-C_18_ column (4.6 mm × 250 mm, Agilent Technologies Co. Ltd., Palo Alto, CA, USA) [25]. Sugar configuration was measured on an Agilent 1260 Infinity HPLC instrument (Agilent Technologies Co. Ltd., Palo Alto, CA, USA) using an Eclipse XDB-C_18_ column (4.6 mm × 250 mm, Agilent Technologies Co. Ltd., Palo Alto, CA, USA) and detected by an Agilent XDB-UV detector (Agilent Technologies Co. Ltd., Palo Alto, CA, USA) [44]. Identification of sugar configuration was done by comparison with retention time of the derivatives of reference sugars, and also comparison with retention time of the corresponding derivatives of the opposite enantiomers.

### 3.5. Structure Analysis

Carboxyl reduction in SPm was carried out according to the method of Taylor and Conrad [45], and the reduction product was designated as SPm-R. Desulfations of the polysaccharides SPm and SPm-R were carried out as previous described [46], and the effectiveness of desulfation procedure was confirmed by determination of residual sulfate in the polysaccharide. The desulfation polysaccharide products of SPm and SPm-R were designated as SPm-Ds and SPm-RDs, respectively. Methylation analysis was performed for the polysaccharides SPm, SPm-Ds, SPm-R and SPm-RDs [47,48]. FTIR spectrum was measured on a Nicolet Nexus 470 spectrometer (Thermo Fisher Scientific, Waltham, MA, USA). NMR spectra were recorded on an Agilent DD2 500 M NMR spectrometer (Agilent Technologies Co. Ltd., Palo Alto, CA, USA). Acetone was used as internal standard (2.225 ppm for ^1^H and 31.07 ppm for ^13^C).

### 3.6. Evaluation of Anticoagulant Activity In Vitro

Anticoagulant activity was assessed by APTT, TT and PT assays [15]. Heparin was used for the comparison of anticoagulant activity of the polysaccharide. Saline solution (0.9% NaCl) was used as control. For the APTT clotting assay, 90 µL of citrated human plasma was mixed with 10 µL of the polysaccharide solutions (0.9% NaCl) at various concentrations (5, 10, 25, 50, 100 µg/mL) and incubated at 37 °C for 60 s. Then, 100 µL of pre-warmed APTT assay reagent was added to 100 µL of mixture and allowed to incubate at 37 °C for 2 min. Thereafter, 100 µL of pre-warmed 0.25 mol/L calcium chloride was added and clotting time was recorded by a SL318 coagulometer (Senlan Medical Science and Trading Co., Ltd., Jinan, China). For the TT clotting assay, 90 µL of citrated human plasma was mixed with 10 µL of the polysaccharide solutions at various concentrations (5, 10, 25, 50, 100 µg/mL) and incubated at 37 °C for 60 s. Then, 200 µL of pre-warmed TT assay reagent was added and clotting time was recorded. For the PT clotting assay, 90 µL of citrated human plasma was mixed with 10 µL of polysaccharide solutions at various concentrations (5, 10, 25, 50, 100 µg/mL) and incubated at 37 °C for 60 s. Then, 200 µL of pre-warmed PT assay reagent was added and clotting time was recorded.

### 3.7. Assessment of Anticoagulant In Vivo and Fibrin(ogen)olytic Properties

Anticoagulant activity in vivo was performed by assays of APTT, TT and PT using rat plasma. Fibrin(ogen)olytic activity was evaluated by assays of D-dimer, PAI-1 and FDP using rat plasma [49,50,51]. Briefly, Sprague-Dawley rats (220–250 g body weight) were randomly divided into six experimental groups (10 rats/group). The experimental rats were anaesthetized with 15% urethane, and then injected with SPm (4, 8, 16 mg/kg), heparin (0.5 mg/kg) or urokinase (20,000 U/kg). The concentration of urokinase referred to the clinical dosage in human according to the specification of the urokinase injection and the related literature [25]. Saline solution was used as control. After 30 min to allow for circulation, the rats were secured in the supine position, and the blood was drawn from the abdominal aorta. APTT, TT and PT clotting assays of the rat plasma were determined using commercial kits according to above mentioned methods. The levels of D-dimer, PAI-1 and FDP in the blood were determined using corresponding commercial kits. D-dimer and FDP assays were performed on a CS-5100 automated blood coagulation analyzer (Sysmex Corporation, Kobe, Japan). PAI-1 assay was performed on a CA-7000 automated blood coagulation analyzer (Sysmex Corporation, Kobe, Japan).

### 3.8. Assay of Thrombolytic Activity In Vitro

Thrombolytic activity in vitro was evaluated by clot lytic rate assays. Clot lytic rate was measured as described by Omura et al. [52]. The blood was taken from the abdominal aorta of Sprague-Dawley rats (220–250 g body weight), and collected in a silicone tube. The blood was placed at room temperature until the big blood clot was completely formed. The clot was rinsed with saline solution, and the liquid in the surface of the clot was removed by filter paper. Then the clot was cut into appropriate pieces, weighed and put into polyethylene tubes, respectively. The tubes were randomly divided into five experimental groups (6 clots/group): SPm (4, 8, 16 mg/mL), urokinase (100 U/mL) and saline solution groups. The appropriate concentration of urokinase was selected by a pre-experiment. Sample (1 mL) was added to the tube, followed by shaking incubation at 85 rpm for 24 h at 37 °C. The residual clot was drawn out from the tube. The liquid in the surface of clot was removed. The wet weight of the residual clot was immediately determined. The clot lytic rate was calculated according to the equation: clot lytic rate (%) = (1 − Wr/W) × 100, where W is the wet weight of the whole clot, and Wr is the wet weight of the residual clot.

### 3.9. Statistical Analysis

Triplicates were used in all the bioassay experiments and the data were expressed as the mean ± standard deviation (SD). Statistical analyses of the data were performed by one-way ANOVA followed by Dunnett’s test (GraphPad Prism 6.00, La Jolla, CA, USA). Statistical significance is denoted by asterisks, where * and ^#^ represented *p* < 0.05, ** and ^##^ represented *p* < 0.01.

## 4. Conclusions

SPm is a novel seaweed polysaccharide with different structural characteristics from previously described polysaccharides from marine algae. SPm consists of →3)-α-l-Rha*p*-(1→ and →2)-α-l-Rha*p*-(1→ residues with sulfation at C-3 or C-2, while small amount of xylose and glucuronic acid exist in the form of β-d-Xyl*p*(4SO_4_)-(1→ and β-d-GlcA-(1→. SPm possessed strong anticoagulant activity in vitro and in vivo, and also exhibited high fibrin(ogen)olytic and thrombolytic activities. SPm may be a promising anticoagulant polysaccharide, and have potential as a drug or a food supplement for health promotion and treatment of thrombotic diseases. Further study on the structure-activity relationship of the sulfated rhamnans from seaweed will aid understanding their anticoagulant activities and may ultimately lead to the development of novel anticoagulant drugs.

## Figures and Tables

**Table 1:** Result of fibrin(ogen)olytic activity assay of SPm.

Sample	Concentration	D-dimer (mg/L)	PAI-1 (U/mL)	FDP (μg/mL)
Control	0 mg/kg	<0.10 ^a^	1.17 ± 0.10	0.50 ± 0.01
SPm ^b^	4 mg/kg	<0.10	0.56 ± 0.11 **	2.34 ± 0.42 **
	8 mg/kg	0.28 ± 0.05 **^##^	0 **^##^	4.67 ± 0.38 **^##^
	16 mg/kg	0.25 ± 0.03 **^##^	0 **^##^	3.94 ± 0.26 **^##^
Urokinase	20,000 U/kg	<0.10	0.62 ± 0.13 **	2.26 ± 0.52 **

**^a^** the D-dimer levels of control, urokinase and SPm at 4 mg/kg groups were below detection limit in this assay. Significance: ** *p* < 0.01 vs. the control group; ^##^ *p* < 0.01 vs. the urokinase group. ^b^ 33.88% of total number of the sugar residues in SPm was substituted by sulfate ester groups.

**Table 2:** Result of thrombolytic activity assay in vitro of SPm.

Sample	Concentration	Clot Lytic Rate (%)
Control	0 mg/mL	6.60 ± 0.14
SPm ^a^	4 mg/mL	12.87 ± 0.28 **
	8 mg/mL	23.47 ± 0.26 **^#^
	16 mg/mL	38.26 ± 0.54 **^##^
Urokinase	100 U/mL	19.92 ± 0.66 **

^a^ 33.88% of total number of the sugar residues in SPm was substituted by sulfate ester groups. Significance: ** *p* < 0.01 vs. the control group; ^#^ *p* < 0.05, ^##^ *p* < 0.01 vs. the urokinase group.

## Supplementary Materials

The following are available online at http://www.mdpi.com/1660-3397/16/7/243/s1, Figure S1: HPGPC chromatogram, HPLC chromatogram and IR spectrum of SPm, Figure S2. NMR spectra of SPm, Table S1: Results of Methylation analysis of SPm, SPm-Ds, SPm-R and SPm-RDs, Table S2: Chemical shifts assignments of NMR spectra of SPm.
