# Supplementary material for: Structural Characteristics and Anticoagulant Property In Vitro and In Vivo of a Seaweed Sulfated Rhamnan
Source: Mar Drugs. 2018 Jul 20;16(7):243. doi: 10.3390/md16070243 (PMC6070894; doi:10.3390/md16070243)
Supplement: Supplementary file 1 [file marinedrugs-16-00243-s001.pdf]

Supplementary Tables

Table S1. Results of Methylation analysis of SPm, SPm-Ds, SPm-R and SPm-RDs.

Methylated alditol acetate	Molar percent ratios				Linkage pattern
	SPm	SPm-Ds	SPm-R	SPm-RDs	
1,2,5-Tri-O-acetyl-3,4-di-O-methyl-rhamnitol	23.70	35.18	21.68	32.20	→2)-Rha*p*-(1→
1,3,5-Tri-O-acetyl-2,4-di-O-methyl-rhamnitol	36.12	52.02	33.52	48.20	→3)-Rha*p*-(1→
1,2,3,5-Tetra-O-acetyl-4-O-methyl-rhamnitol	33.68	6.30	30.92	5.72	→2,3)-Rha*p*-(1→
1,5-Di-O-acetyl-2,3,4,6-tetra-O-methyl-glucitol	nd [a]	nd [a]	7.86	7.86	Glc*p*-(1→
1,5-Di-O-acetyl-2,3,4-tri-O-methyl-xylitol	nd [a]	6.50	nd [a]	6.02	Xyl*p*-(1→
1,4,5-Tri-O-acetyl-2,3-di-O-methyl-xylitol	6.50	nd [a]	6.02	nd [a]	→4)-Xyl*p*-(1→

[a] Not detected

Table S2. Chemical shifts assignments of NMR spectra of SPm.

Sugar residues	Chemical shifts (ppm)					
	H1/C1	H2/C2	H3/C3	H4/C4	H5/C5	H6/C6
A：→3)-Rha*p*(2SO_4)-(1→	5.49/100.74	4.72/78.42	4.10/78.42	3.66/73.29	3.81/70.81	1.38/18.36
B：→2)-Rha*p*(3SO_4)-(1→	5.34/100.74	4.30/80.84	4.55/79.00	3.66/73.29	3.81/70.81	1.38/18.36
C：→2,3)-Rha*p*-(1→	5.29/100.74	4.30/80.84	4.10/78.42	3.60/73.29	3.81/70.81	1.38/18.36
D：→2)-Rha*p*-(1→	5.26/100.74	4.30/80.84	3.96/70.81	3.60/73.29	3.81/70.81	1.38/18.36
E：→3)-Rha*p*-(1→	5.08/103.32	4.20/70.81	3.96/78.42	3.66/73.29	3.81/70.81	1.38/18.36

Supplementary Figure Legends

Figure S1. HPGPC chromatogram, HPLC chromatogram and IR spectrum of SPm. (a) HPGPC chromatogram of SPm on a Shodex OHpak SB-804 HQ column and the standard curve of molecular weight. (b) HPLC chromatogram for monosaccharide composition analysis of SPm (Man: D-mannose, GlcN: D-glucosamine, Rha: L-rhamnose, GlcA: D-glucuronic acid, GalA: D-galacturonic acid, Glc: D-glucose, Gal: D-galactose, Xyl: D-xylose, Ara: L-arabinose, Fuc: L-fucose). (c) IR spectrum of SPm.

Figure S2. NMR spectra of SPm. (a) ^{1}H NMR spectrum. (b) ^{13}C NMR spectrum. (c) ^{1}H–^{1}H COSY spectrum. (d) ^{1}H–^{13}C HSQC spectrum. (e) ^{1}H–^{1}H NOESY. A–E correspond to →3)-α-L-Rha*p*(2SO_4)-(1→, →2)-α-L-Rha*p*(3SO_4)-(1→, →2,3)-α-L-Rha*p*-(1→, →2)-α-L-Rha*p*-(1→ and →3)-α-L-Rha*p*-(1→, respectively. Rha*p*: rhamnopyranose.

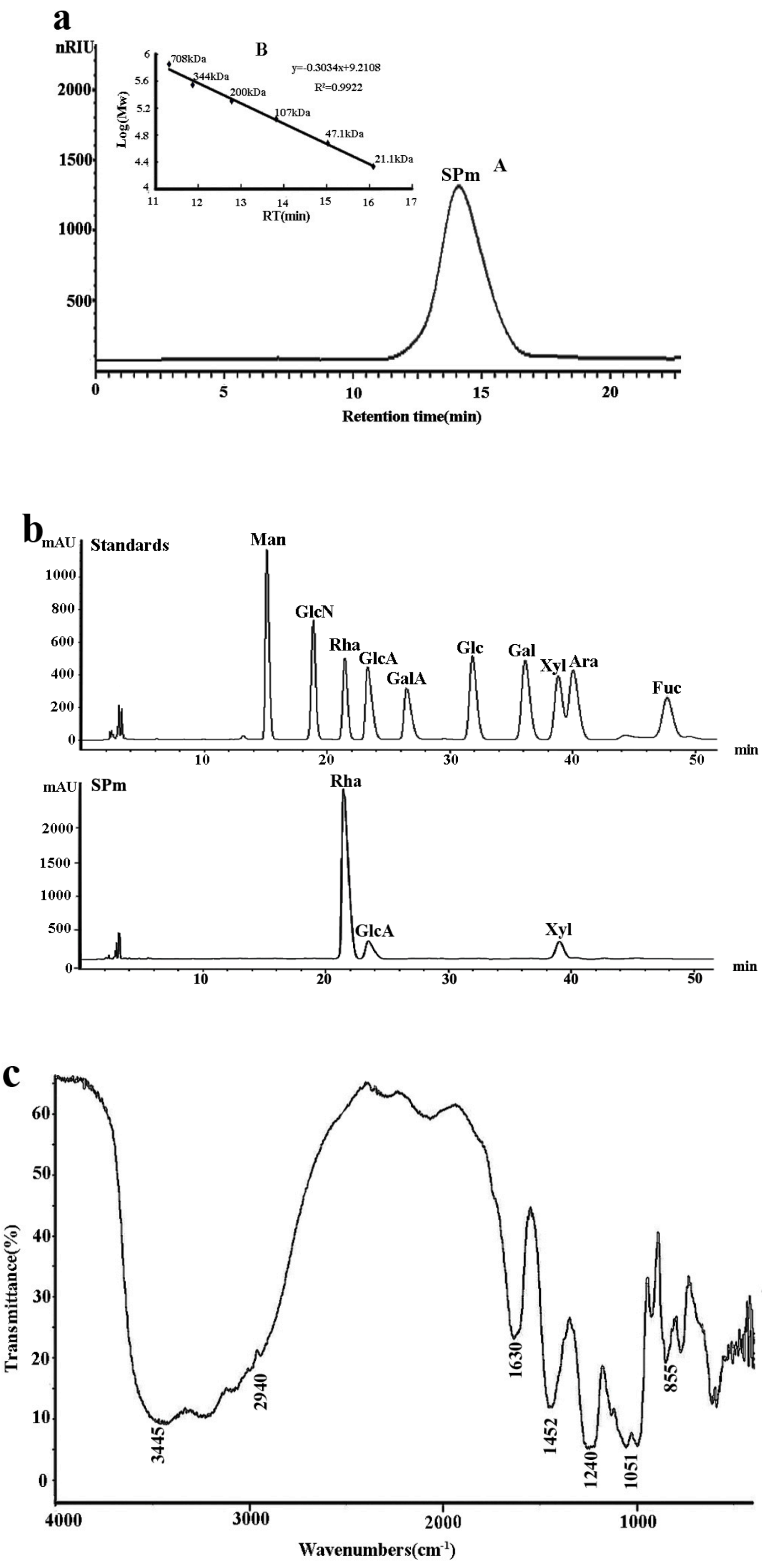
a
nRIU
B
708kDa
344kDa
200kDa
107kDa
47.1kDa
21.1kDa
y=-0.3034x+9.2108
R²=0.9922
Log(Mw)
RT(min)
SPm
A
Retention time(min)
b
mAU
Standards
Man
GlcN
Rha
GlcA
GalA
Glc
Gal
Xyl
Ara
Fuc
min
mAU
SPm
Rha
GlcA
Xyl
min
c
Transmittance(%)
3445
2940
1630
1452
1240
1051
855
Wavenumbers(cm-1)

Figure S1

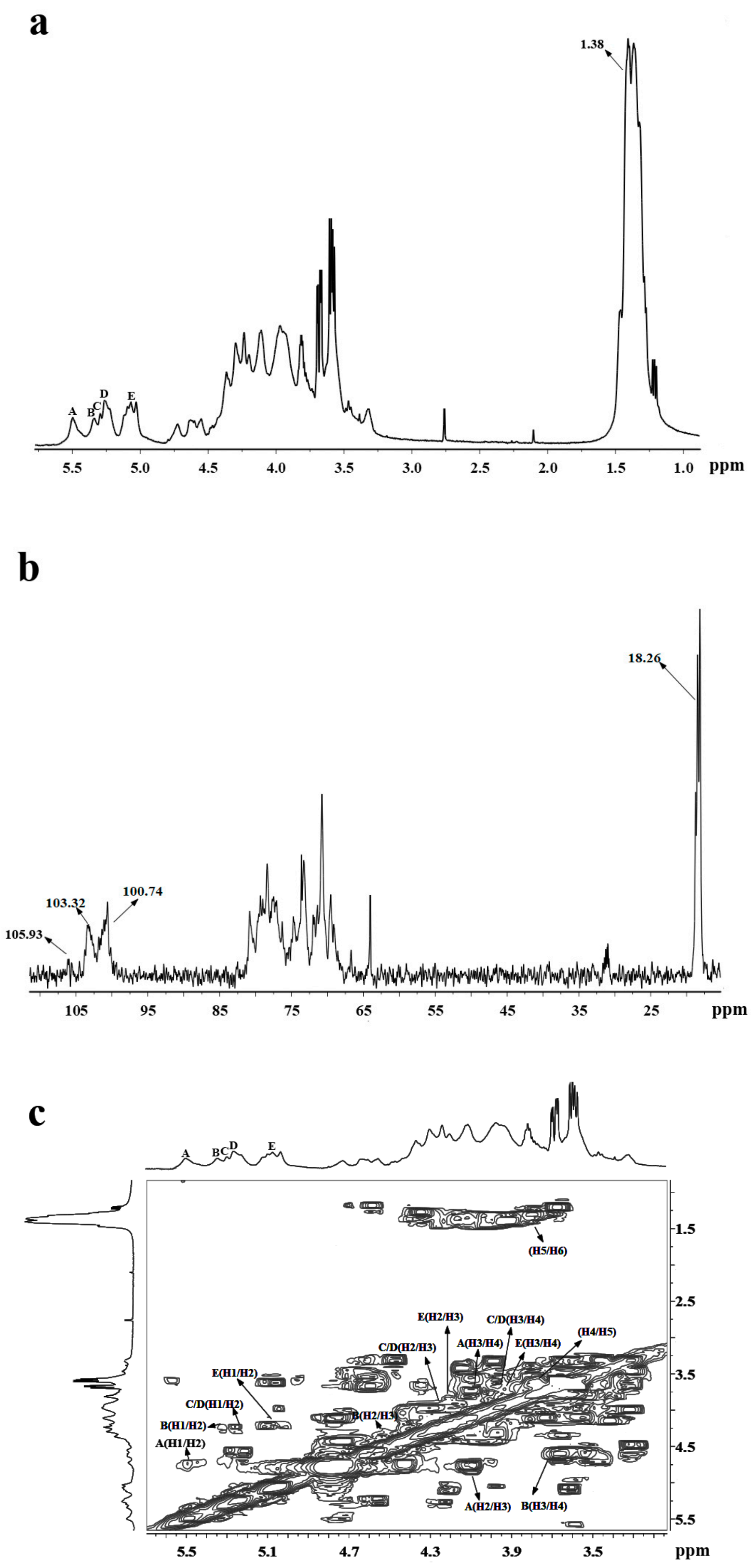

a
1.38
A
B
C
D
E
5.5
5.0
4.5
4.0
3.5
3.0
2.5
2.0
1.5
1.0
ppm
b
18.26
103.32
100.74
105.93
105
95
85
75
65
55
45
35
25
ppm
c
A
BC
D
E
(H5/H6)
E(H2/H3)
C/D(H3/H4)
C/D(H2/H3)
A(H3/H4)
E(H3/H4)
(H4/H5)
E(H1/H2)
C/D(H1/H2)
B(H2/H3)
B(H1/H2)
A(H1/H2)
A(H2/H3)
B(H3/H4)
1.5
2.5
3.5
4.5
5.5
5.5
5.1
4.7
4.3
3.9
3.5
ppm

d

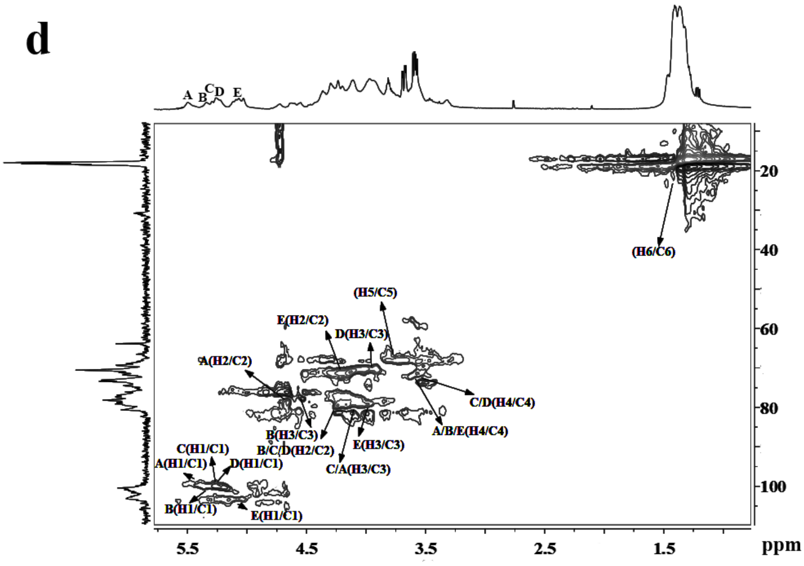

A B C D E
(H6/C6)
(H5/C5)
E(H2/C2)
D(H3/C3)
A(H2/C2)
C/D(H4/C4)
A/B/E(H4/C4)
B(H3/C3)
B/C/D(H2/C2)
E(H3/C3)
C/A(H3/C3)
C(H1/C1)
A(H1/C1)
D(H1/C1)
B(H1/C1)
E(H1/C1)
20
40
60
80
100
5.5
4.5
3.5
2.5
1.5
ppm

e

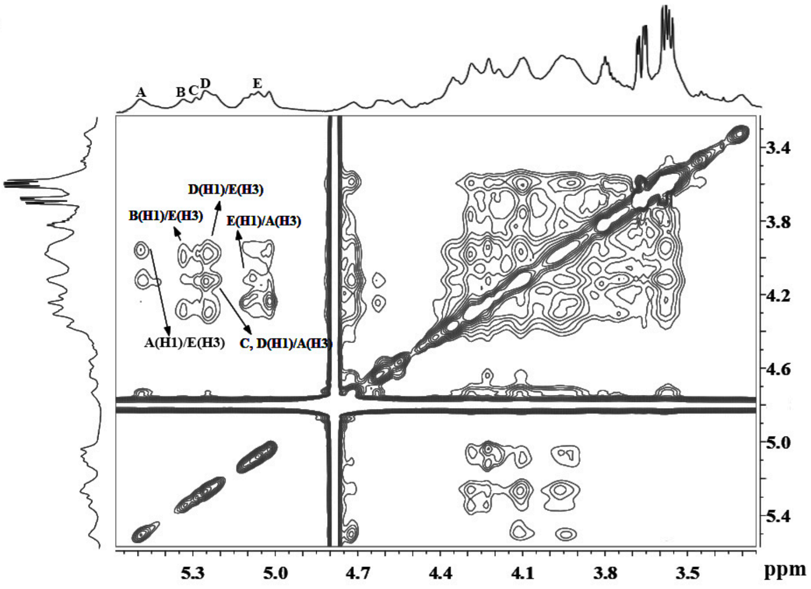

A B C D E
D(H1)/E(H3)
B(H1)/E(H3)
E(H1)/A(H3)
A(H1)/E(H3)
C, D(H1)/A(H3)
3.4
3.8
4.2
4.6
5.0
5.4
5.3
5.0
4.7
4.4
4.1
3.8
3.5
ppm

Figure S2
